# Full-Genome Sequence of Influenza A(H5N8) Virus in Poultry Linked to Sequences of Strains from Asia, the Netherlands, 2014

**DOI:** 10.3201/eid2105.141839

**Published:** 2015-05

**Authors:** Ruth Bouwstra, Rene Heutink, Alex Bossers, Frank Harders, Guus Koch, Armin Elbers

**Affiliations:** Wageningen University and Research Centre, Central Veterinary Institute, Lelystad, the Netherlands

**Keywords:** influenza, H5N8, poultry, the Netherlands, genome sequencing, viruses, Asian strains

## Abstract

Genetic analyses of highly pathogenic avian influenza A(H5N8) virus from the Netherlands, and comparison with strains from Europe, South Korea, and Japan, showed a close relation. Data suggest the strains were probably carried to the Netherlands by migratory wild birds from Asia, possibly through overlapping flyways and common breeding sites in Siberia.

Highly pathogenic avian influenza (HPAI) A(H5N8) virus probably originated in China, where it was isolated in 2009–2010 (*1*). Pathogenicity studies showed that the virus was highly virulent in chickens but mildly or moderately virulent in wild ducks. Phylogenetic research demonstrated that it was the product of various reassortment events: the virus’s RNA consists of segments that come from other influenza viruses. The backbone of the HPAI (H5N8) virus is formed by parts of the HPAI (H5N1) virus that has circulated in China since 1997 and spread worldwide since 2004. 

Beginning in January 2014, H5N8 virus spread rapidly in South Korea, initially mainly among farmed ducks. During the first outbreaks among farmed ducks, numerous dead Baikal teals (*Anas formosa*, a species of migratory wild ducks) were found near the affected farms, leading to the hypothesis that infection was carried by the wild ducks. Genetic analysis of the virus indicated that isolates from infected domesticated ducks and dead Baikal teals in the surrounding area in South Korea strongly resembled earlier isolates from China (*2*). The analysis also indicated that the HPAI (H5N8) virus in South Korea is a product of reassortment of A/duck/Jiangsu/k1203/2010 (H5N8) and other avian influenza viruses that co-circulated among birds in East Asia during 2009–2012 (*3*). Kang et al. (*4*) recently demonstrated by experimental infection of wild ducks (*A. platyrhynchos*) and Baikal teals that HPAI (H5N1) and (H5N8) virus isolates did not cause serious illness or death in these species. Recent phylogenetic studies of HPAI (H5H8) viruses isolated from infected poultry and wild birds in 2014 in South Korea indicate that migrating birds played a key role in the introduction and spread of the virus in the initial phase of the 2014 outbreak (*5*). In mid-April 2014, the presence of HPAI (H5N8) virus was demonstrated at a poultry farm in Japan after a rise in the death rate was noted (*6*). During a monitoring program in November 2014, fecal samples of migrating Bewick’s Tundra swans (*Cygnus columbianus bewickii*) tested positive for the HPAI (H5N8) virus. We conducted full-length sequencing to elucidate the origin of the HPAI (H5N8) virus detected in the Netherlands.

## The Study

On November 9, 2014, chickens in 1 of 6 poultry houses on a 124,000-bird indoor-layer farm in the Netherlands began dying at an exponentially increasing rate. The dead chickens were submitted for necropsy to the Dutch Animal Health Service (http://www.gdanimalhealth.com) on November 14. RNA was extracted from cloacal and oropharyngeal samples from clinically affected hens with positive results from the screening influenza real-time reverse transcription PCR (*7*), which detects all avian influenza virus subtypes. As a standard procedure, the swab samples were forwarded to the Central Veterinary Institute, the Netherlands’ national reference laboratory. Positive screening samples were checked for the presence of H5 and H7 influenza subtypes by real-time reverse transcription quantitative PCR as recommended by the European Union reference laboratory. Hemagglutinin (HA) and neuraminidase (NA) sequence analysis was performed by using PCR fragments that were generated according to previously described protocols (*8**,**9*). The HA cleavage site showed polybasic properties RNSPLRERRRKR*GLFGAIA, confirming the high pathogenicity of the virus. In addition, HA and NA sequence results showed that the virus subtype was H5N8.

At the start of the outbreak, preliminary sequencing of the cleavage site showed that it shared high similarity with that of the outbreak strain from Germany. However, complete sequencing was necessary for an investigation of the origin and emergence of this virus in Europe, specifically in the Netherlands. Therefore, we amplified all 8 RNA genome segments of the outbreak virus by using universal 8-segment primers and then directly sequenced the segments (*10*). Purified amplicons were sequenced at high coverage (average >1,000) by using the Nextera library preparation method and MiSeq system (Illumina, San Diego, CA, USA), generating paired-end read lengths of 150 bases. High-quality quality control–passed sequence reads were iteratively mapped by using Bowtie 2 (*11*) against the genome sequence of the H5N8 virus from South Korea (GenBank accession nos. KJ511809–KJ511816) to generate a majority (>80% evidence) consensus sequence of all segments. The consensus sequences were compared with de novo–assembled sequence reads by using SPAdes version 3 (*12*); substantial differences were not detected. Most consensus sequences were submitted to the Global Initiative on Sharing Avian Influenza Data (accession no. EPI_ISL_167905). We subsequently performed a molecular phylogenetic analysis on all nucleic acid sequences by using the maximum-likelihood method based on the Tamura-Nei model in MEGA6.0 (*13*). Accession numbers and sequence providers are listed in the Technical Appendix.

Genetic analysis shows that the H5N8 virus from the Netherlands (A/chicken/Netherlands/14015526/2014) and viruses from poultry and wild birds from Europe and 2 strains from Japan (A/duck/Chiba/26-372-48/2014 and A/duck/Chiba/26-372-61/2014) detected thereafter are closely related. Analysis also showed that the viruses are descendants of 3 strains isolated in early 2014: A/broiler duck/Buan2/2014 and A/Baikal teal/Korea/Donglim3/2014 from South Korea and A/chicken/Kumamoto/1-7/2014 from Japan. HPAI (H5N8) virus isolated from a wigeon (*Anas penelope*) in Sakha, northeastern Russia, is a precursor phylogenetically located at the node of European and Chiba viruses (Figure); this virus was isolated in September 2014, but the sequence was released in December 2014 (*15*).

**Figure:**
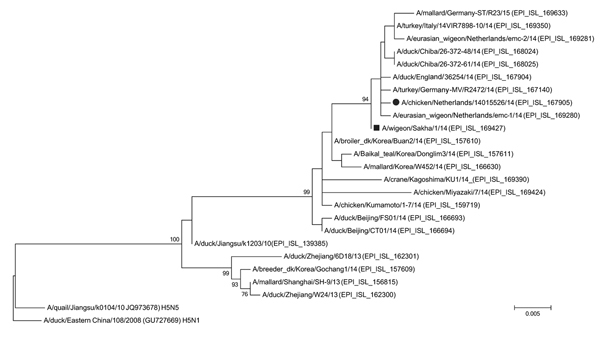
Phylogenetic tree of hemagglutinin gene of highly pathogenic avian influenza A(H5N8) viruses. The evolutionary history was inferred by using the maximum-likelihood method based on the Tamura-Nei model in MEGA6 (*14*). The tree with the highest log likelihood is shown. The percentage of trees in which the associated taxa clustered together is shown next to the branches. Initial tree(s) for the heuristic search were obtained automatically by applying neighbor-joining and BIONJ (*15*) algorithms to a matrix of pairwise distances estimated by using the maximum composite likelihood approach and then selecting the topology with superior log likelihood value. The Tamura-Nei model was used by assuming a gamma distributed rate among nucleotide sites. The tree is drawn to scale; scale bar indicates the number of nucleotide substitutions per site. The analysis involved 25 nt sequences. All positions containing gaps and missing data were eliminated. There were a total of 761 nt positions in the final dataset. Black dot indicates A/chicken/Netherlands/14015526/2014; black square indicates A/wigeon/Sakha/1/2014.

We determined the number of per site base substitutions between the sequences by using the maximum composite likelihood in MEGA6 (*13*). Sequences of the 8 genome segments in the H5N8 virus from the Netherlands differed from those of strains A/broiler duck/Korea/Buan2/2014, A/Baikal teal/Korea/Donglim3/2014, and A/chicken/Kumamoto/1-7/2014 by a minimum of 0 and a maximum of 0.009 substitutions. On the basis of data in the National Center for Biotechnology Information Influenza Virus Resource (http://www.ncbi.nlm.nih.gov/genomes/FLU/FLU.html) and EpiFlu (http://www.gisaid.org) databases, the H5N8 virus from the Netherlands shares the highest similarity with strain A/Baikal teal/Korea/Donglim3/2014 from South Korea.

## Conclusions

Genetic analysis of influenza A(H5N8) virus from the Netherlands indicates that the virus probably was spread by migratory wild birds from Asia, possibly through overlapping flyways and common breeding sites in Siberia. In addition to the outbreak in the Netherlands, several other outbreaks of HPAI (H5N8) virus infections were reported in Europe at the end of 2014 after exponentially increasing deaths occurred in chicken and turkey flocks. Genetic sequences submitted to the EpiFlu database indicated that the viruses from Europe showed a strong similarity to viruses isolated earlier in 2014 in South Korea, China, and Japan. An H5N8 virus isolated from a wigeon in Russia in September 2014 is located in the phylogenetic tree near the node of all sequences for H5N8 viruses from Europe. In regard to time, this location fits the hypothesized route of H5N8 virus introduction into Europe. Furthermore, for several reasons, it is highly likely that the introduction of HPAI (H5N8) virus into the indoor-layer farm in the Netherlands occurred via indirect contact. First, despite intensive monitoring, H5N8 viruses have never been detected in commercial poultry or wild birds in the Netherlands. Second, when the virus was detected, the Netherlands had no direct trade contact with other European countries or Asia that might explain a route of introduction. Third, because of the severity of disease in galliforms, outbreaks of H5N8 in the Netherlands before November 2014 would have been noticed.

Technical Appendix Sequences of the H5N8 virus isolates from the Netherlands were compared with sequences obtained from the Global Initiative on Sharing Avian Influenza Data’s EpiFlu Database.
